# Predisposing factors to acquisition of acute respiratory tract infections in the community: a systematic review and meta-analysis

**DOI:** 10.1186/s12879-021-06954-3

**Published:** 2021-12-14

**Authors:** Ashley Hammond, Alice Halliday, Hannah V. Thornton, Alastair D. Hay

**Affiliations:** 1 Centre for Academic Primary Care, NIHR School for Primary Care Research, Bristol Medical School, University of Bristol, Canynge Hall, 39 Whatley Road, Bristol, BS8 2PS UK; 2 School of Cellular and Molecular Medicine, Biomedical Sciences Building, University of Bristol, University Walk, Bristol, BS8 1TD UK

**Keywords:** Respiratory tract infections, Community, Predisposing factors

## Abstract

**Background:**

Preventing respiratory tract infections (RTIs) could have profound effects on quality of life, primary care workload, antibiotic prescribing and stewardship. We aimed to identify factors that increase and decrease RTI acquisition within Organisation for Economic Cooperation and Development (OECD) member countries.

**Methods:**

Systematic search of Medline, Embase, Cochrane and ISI Web of Knowledge for studies conducted up to July 2020 reporting predisposing factors for community RTI acquisition. Pooled odds ratios were calculated using a random-effects model.

**Results:**

23 studies investigated risk factors associated with community-acquired pneumonia (n = 15); any RTI (n = 4); influenza like illness (n = 2); and lower RTI (n = 2). Demographic, lifestyle and social factors were: underweight BMI (pooled odds ratio (OR_p_ 2.14, 95% CI 1.58 to 2.70, p = 0.97); male sex (OR_p_ 1.30, 95% CI 1.27 to 1.33, p = 0.66); contact with pets (OR_p_ 1.35, 95% CI 1.16 to 1.54, p = 0.72); contact with children (OR_p_ 1.35, 95% CI 1.15 to 1.56, p = 0.05); and ex-smoking status (OR_p_ 1.57, 95% CI 1.26 to 1.88, p = 0.76). Health-related factors were: chronic liver condition (OR_p_ 1.30, 95% CI 1.09 to 1.50, p = 0.34); chronic renal condition (OR_p_ 1.47, 95% CI 1.09 to 1.85, p = 0.14); and any hospitalisation in previous five years (OR_p_ 1.64, 95% CI 1.46 to 1.82, p = 0.66).

**Conclusions:**

We identified several modifiable risk factors associated with increased likelihood of acquiring RTIs in the community, including low BMI, contact with children and pets. Modification of risk factors and increased awareness of vulnerable groups could reduce morbidity, mortality and antibiotic use associated with RTIs.

***PROSPERO registration*:**

CRD42019134176.

**Supplementary Information:**

The online version contains supplementary material available at 10.1186/s12879-021-06954-3.

## Background

Respiratory tract infections (RTIs) are the single most common infections seen in primary care, and a major contributor to the overall burden of disease worldwide. In the UK, respiratory illnesses cost the NHS around £3 billion annually, including £1.7 billion in morbidity costs such as hospital admissions and antibiotic prescriptions [1]. RTIs are the reason for between 300 and 400 general practice consultations per 1000 registered patients per annum [2], and can cause considerable morbidity to patients including loss of earnings due to sickness absence [3]. In 2016 it was reported that coughs and colds were the most common reasons for sickness absence in the UK, accounting for 34 million days lost [4]. Globally, respiratory infections cause considerable burden; in 2021 the Global Burden of Disease study reported that upper respiratory infection incidence accounted for 42.83% of cases from all causes between 1990 and 2019, and highlighted the need for further research in the prevention and treatment of respiratory infections worldwide [5].

On average, adults will have between two and five RTIs annually, usually the common cold or upper respiratory infections [6]. RTIs are also the most common indications for antibiotic prescribing [7], accounting for around 60% of all antibiotics prescribed in primary care, despite rarely providing patient benefit [8–10].

We are currently in the midst of a global coronavirus (COVID-19) pandemic, a respiratory virus, responsible for more than 1 million deaths worldwide [11]. There is a need to explore the ways in which RTIs can be prevented, thus reducing the number of consultations in primary care, avoid potentially unnecessary antibiotic use, and to be better prepared for future respiratory pandemics.

This systematic review and meta-analysis aims to identify the factors increasing and decreasing the risk of acquiring respiratory infections in communities within OECD member countries. Their identification could allow targeting of interventions to reduce the burden of RTIs and other common community-acquired infections.

## Methods

### Search strategy and selection criteria

We searched Medline, Embase, Cochrane and ISI Web of Knowledge for articles published in any language between January 1946 and September 2020. Medline and Embase were searched using Ovid interrogation software. MeSH terms for these databases included “respiratory tract infections”, “community-acquired infections” and “primary health care”. MeSH terms were combined with text word searches which included “cough”, “pneumonia”, “chest infection”, “influenza”, “acute illness”, “family practice” and “risk factors”. Grey and unpublished literature was searched for using ISI Web of Knowledge software and included journal articles, patents, websites, conference proceedings, government and national reports and open access material. Reference lists of selected key papers were screened and authors who appeared multiple times were contacted to request details of any relevant published or unpublished work. All full-text papers were subject to citation searches. See Additional file 1: Table S1 for full search strategy. We chose not to include studies related specifically to COVID-19, as we feel this warrants a separate review once more data on COVID-19 are available.

One reviewer screened all titles and abstracts, which were then subject to a double-screen check by a second reviewer. Population, Intervention, Comparator, Outcome and Study design (PICOS) criteria were used for inclusion and exclusion decisions, outlined in Additional file 1: Table S2. Studies were eligible for inclusion if they were quantitative and reporting individual or population-level identified risk factors for acquiring a RTI in the community. All eligible studies were conducted in an Organisation for Economic Co-operation and Development (OECD) member country as there is some variation in risk of acquiring RTIs in less developed, non-OECD countries which would make it difficult to compare our findings [12]. To avoid duplication of work, studies which had been included in a previous systematic review conducted within the last 10 years which investigated one specific predisposing factor only to RTI acquisition, such as smoking, were excluded.

### Data extraction and quality assessment

Full text papers for all eligible studies were obtained and data extracted by two independent reviewers (Hammond, Halliday) using a purpose-built spreadsheet, with each reviewer blinded to the others screening/data extraction. Where provided, the following information was extracted for all papers: author, journal, year of publication, study design, study country, study participants, setting and recruitment methods, study time period and age range. Risk factor data was also extracted, including raw data (where available), crude and adjusted odds ratios and their 95% confidence intervals and p-values, where reported.

For all observational studies, the Critical Appraisal Skills Programme (CASP) checklist was used to assess study quality (www.casp-uk.net). Our key quality criteria for eligibility were clear reporting of RTIs as community-acquired within the paper, i.e. the infection was not acquired in a hospital setting, and clear and reliable measures of risk factors including appropriate adjustment for confounders, such as age, sex and comorbidities. A risk of bias score of “high”, “medium” or “low” was applied to each criterion.

### Data synthesis and analysis

Where the same factors were reported in two or more studies, pooled odds ratios were calculated using a random-effects meta-analysis. Crude estimates or raw data were used where available, as adjusted estimates likely take into account different factors, and so are not appropriate to pool. Pooled crude odds ratios were compared with adjusted odds ratios for each factor, where reported in each study. For each pooled analysis, the reference group is those without the specified risk factor. Heterogeneity was assessed using the I^2^ statistic (with corresponding p-values) in line with Cochrane recommendations [13]. An I^2^ of 25%, 50% and 75% was used to signify low level, moderate level and high level heterogeneity. Where heterogeneity was high (≥ 75%), pooled effect estimates were not calculated. A funnel plot was used to investigate publication bias in the studies included in our meta-analysis where possible. Where the factors reported were not the same in two or more studies, estimates were not pooled. Instead, we reported these study results individually only. All statistical analyses were conducted using Stata version 15 software, and all methods undertaken according to PRISMA guidelines [14].

### Patient and public involvement

No patients or members of the public were involved in setting the research question or outcome measures, nor were they involved in developing plans for design, or implementation of the study. No patients or members of the public were asked to advise on interpretation or writing up of results.

## Results

**Fig. 1:**
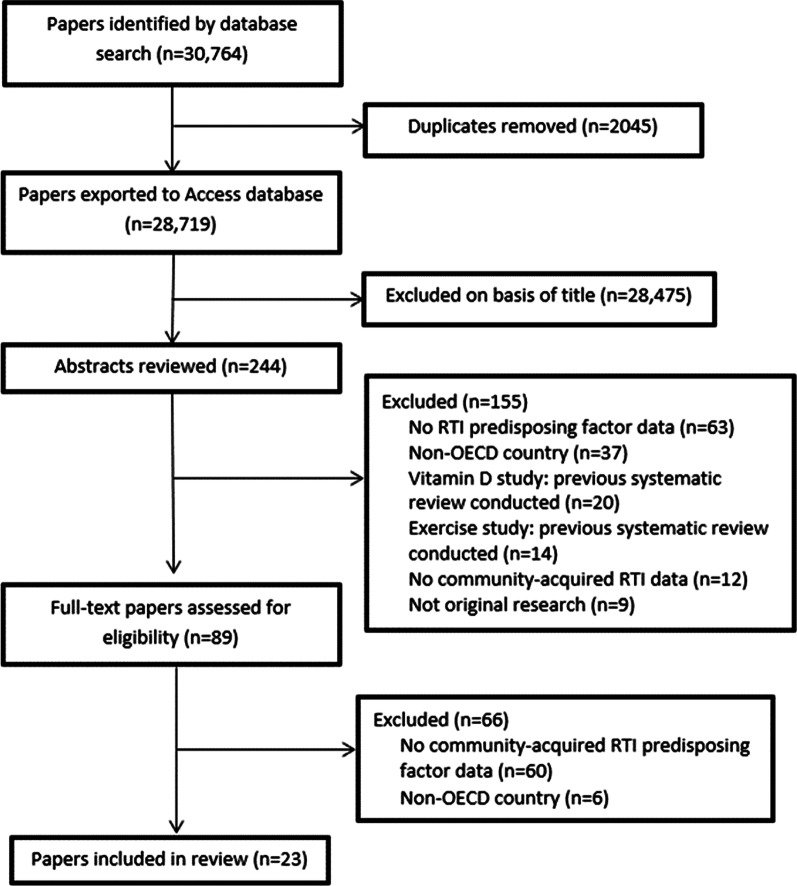
PRISMA flowchart for inclusion and exclusion of studies

Our search strategy returned 28,719 papers in total (see Fig. 1). Following review of titles and abstracts 28,475 papers were excluded. Based on full-text review of 89 papers, 66 were excluded for the following reasons: 60 had no community-acquired RTI risk factor data, and six were conducted in a non-OECD country. Several papers were excluded from our abstract screen due to the fact that previous systematic reviews had been conducted; this was the case for studies investigating the role of either Vitamin D [15, 16], or exercise in the acquisition of respiratory tract infections [17]. We did not find any additional papers on either of these topics which had not been included in the previous systematic reviews. We are also conducting a subsequent review exploring interventions to reduce incidence of infections in the community (PROSPERO ID CRD42021291929), therefore any study exploring the effectiveness of an intervention was not included in this review. This left 23 studies investigating factors increasing and decreasing the risk of acquiring respiratory infections in the community included in the review.

### Study characteristics

We included 23 observational risk factor studies in our review; five studies included children only (< 18 years), nine studies included adults only (18 + years), and nine studies included mixed adult and child populations. Studies were conducted in the USA (n = 6), UK (n = 4), Spain (n = 4), Germany (n = 3), Netherlands (n = 2), Sweden (n = 1), Finland (n = 1), Denmark (n = 1), and New Zealand (n = 1). All extracted study data can be found in Additional file 1: Table S3. Studies reported risk factors for the acquisition of community-acquired pneumonia (n = 15), influenza-like illness (n = 2), lower respiratory tract infection (n = 2), and any RTI (n = 4). All characteristics for included risk factor studies are shown in Table 1.

**Table 1:** Study characteristics

Study characteristics	Number of papers	References
Study design		
Observational	12	[18–29]
Case–control	10	[30–39]
Cross-sectional	1	[40]
Participant age		
Adults (≥ 18 years)	9	[18, 22, 24, 25, 27, 30, 32, 35, 40]
Children (< 18 years)	5	[20, 23, 29, 33, 38]
Mixed adults and children	9	[19, 21, 26, 28, 31, 34, 36, 37, 39]
Recruitment location		
Primary care	14	[19, 20, 22, 23, 25, 26, 28, 31, 32, 34, 36–39]
Outpatient/inpatient	3	[24, 27, 35]
Emergency department	3	[18, 30, 33]
Community	3	[21, 29, 40]
Number of study participants		
< 500	3	[28, 29, 38]
500–1000	2	[37, 39]
1001–2000	7	[20, 26, 27, 30, 31, 33, 40]
2001–5000	3	[32, 35, 36]
5001–10,000	1	[21]
> 10,000	7	[18, 19, 22–25, 34]
Primary RTI investigated		
Community-acquired pneumonia	15	[18, 22, 25–28, 30–38]
Influenza-like-illness	2	[21, 24]
Lower RTI	2	[20, 23]
Any RTI	4	[19, 29, 39, 40]

Most studies had a low risk of bias; the main reason for studies with a medium risk of bias was lack of controlling for confounding factors, unclear reporting of baseline data, or reporting outputs from statistical tests only and not the raw study data. Quality assessment charts are shown in Additional file 2: Fig. S1.

Of the 23 studies included, 12 studies reported crude odds ratio estimates for risk factors which they investigated and were included in a meta-analysis. Adjusted odds ratios could not be pooled due to differences in the risk factors adjusted for in each study. For those studies which did not meet the requirements for meta-analysis, most were due to high heterogeneity (I^2^ > 75%), or reported findings in a different unit of measurement including hazard ratios [19], incidence rate ratios [22], and risk ratios [24, 25], which could not be pooled together with studies reporting odds ratios. Several studies reported factors for which there was no other comparable data in any other studies for meta-analysis. Additional file 1: Table S3 outlines the study characteristics for all papers included in this review, including what data were available.

For reporting purposes, the factors reported by each study were grouped into one of five different categories: (i) demographics; (ii) environmental; (iii) lifestyle; (iv) social, and (v) health. The following five sections summarise the reported factors which were not pooled together by meta-analysis.

### Demographics

There was some evidence that older age increases the likelihood of acquiring an RTI, though studies investigating age varied in their reporting. One study used Townsend scores to measure deprivation, and found evidence that increasing deprivation increases the odds of acquiring CAP [34]. There was considerable variation in the way that education was reported by different studies, which made it difficult to compare any two studies. There was mixed evidence of an association between low education levels, however Gessner et al. (2009) appears to report a biological gradient, where risk of lower RTI acquisition increases as years of formal education decreases [23]. No studies reported evidence that employment status, marital status, rural versus urban living or income was associated with RTI acquisition. Only one study in New Zealand reported any data on race and ethnicity. Additional file 2: Fig. S2 summarises the demographic factors investigated in the included studies, where studies are grouped based on the factors they reported.

### Environmental and occupational

One study investigated household proximity to animal farms as a risk factor for RTI acquisition but found no clear associations [30]. Another study in children under 4 years reported an association between fewer than 30 min daily outside time in previous month and RTI acquisition (crude OR 2.34, 95% CI 1.42 to 3.88) [33]. One study investigated work exposures as risk factors for CAP acquisition and reported an association between work related contact with animals, excrements and viscera and CAP acquisition (crude OR 1.29, 95% CI 1.02 to 1.62), and sudden temperature changes at work in previous 3 months (including refrigerators, furnace or kitchen) and CAP acquisition (crude OR 3.12, 95% CI 2.16 to 4.50) [36] Additional file 2: Fig. S3 summarises the environmental risk factors investigated in the included studies.

### Lifestyle

Smoking, either current of former smoker was associated with acquisition of both ILI and CAP based on crude OR from two studies [21, 35]. One study reported an association between children who were not breastfed and increased likelihood of CAP acquisition (crude OR 2.72, 95% CI 1.52 to 4.87), however the same study did not find associations between exclusive breastfeeding and reduced likelihood of CAP acquisition [33]. Four studies reported risk factors as either hazard ratios, incidence rate ratios or risk ratios as opposed to odds ratios and so were reported separately (see Additional file 2: Fig. S4). An underweight BMI was associated with an increased likelihood of CAP acquisition from two studies, one reported a hazard ratio of 1.33 (95% CI 1.04 to 1.70) [19] and the other reported an incidence rate ratio of 2.62 (95% CI 2.10 to 3.24) [22]. No associations were reported for multivitamin intake and increased likelihood of RTI acquisition. Additional file 2: Fig. S5 summarises the lifestyle-related risk factors investigated in the included studies.

### Social

Only one study reported an association with acquiring a RTI, CAP specifically, and that was having more than 10 people living in the household [36]. One population-based study reported a risk ratio of the effect of a percentage increase in the paediatric population on the rate of adult acute RTIs (per 1000 population), where the paediatric population was defined as all children aged 0–17 years (see Additional file 2: Fig. S2) [24]. The crude risk ratio reported was 1.04 (95% CI 1.02 to 1.05), suggesting that as the paediatric population increases so does the rate of adult (≥ 18 years) RTIs. Additional file 2: Fig. S6 summarises the social risk factors investigated in the included studies.

### Comorbidity and medical history

Due to the large number of different health-related risk factors that were reported in our included studies, we split these into two groups: current health conditions and medical history. Additional file 2: Fig. S7 summarises the health condition-related risk factors investigated in the included studies. One study investigated several chronic conditions as risk factors, and reported associations with CAP acquisition and multiple chronic conditions (cardiovascular, cerebrovascular, rheumatoid and immunosuppressive) [18]. There was evidence from several studies that heart disease is associated with an increased odds of acquiring a RTI, however heterogeneity between the studies was too high for them to be meta-analysed.

Several studies reported immunisation-related risk factors, including having previously received an influenza vaccination, however heterogeneity between these studies was too high for meta-analysis. Several studies reported previous CAP and previous RTIs of any kind, but again high heterogeneity prevented inclusion in our meta-analysis. All studies reporting previous CAP episodes reported an association with increased likelihood of acquiring community-acquired CAP. One study investigated recent dental visits as a risk factor, and found that visiting the dentist in the past month had a protective effect (crude OR 0.71, 95% CI 0.55 to 0.92). Additional file 2: Fig. S8 shows the medical history-related risk factors for RTI acquisition in the included studies.

### Meta-analysis of RTI risk factor studies

**Fig. 2:**
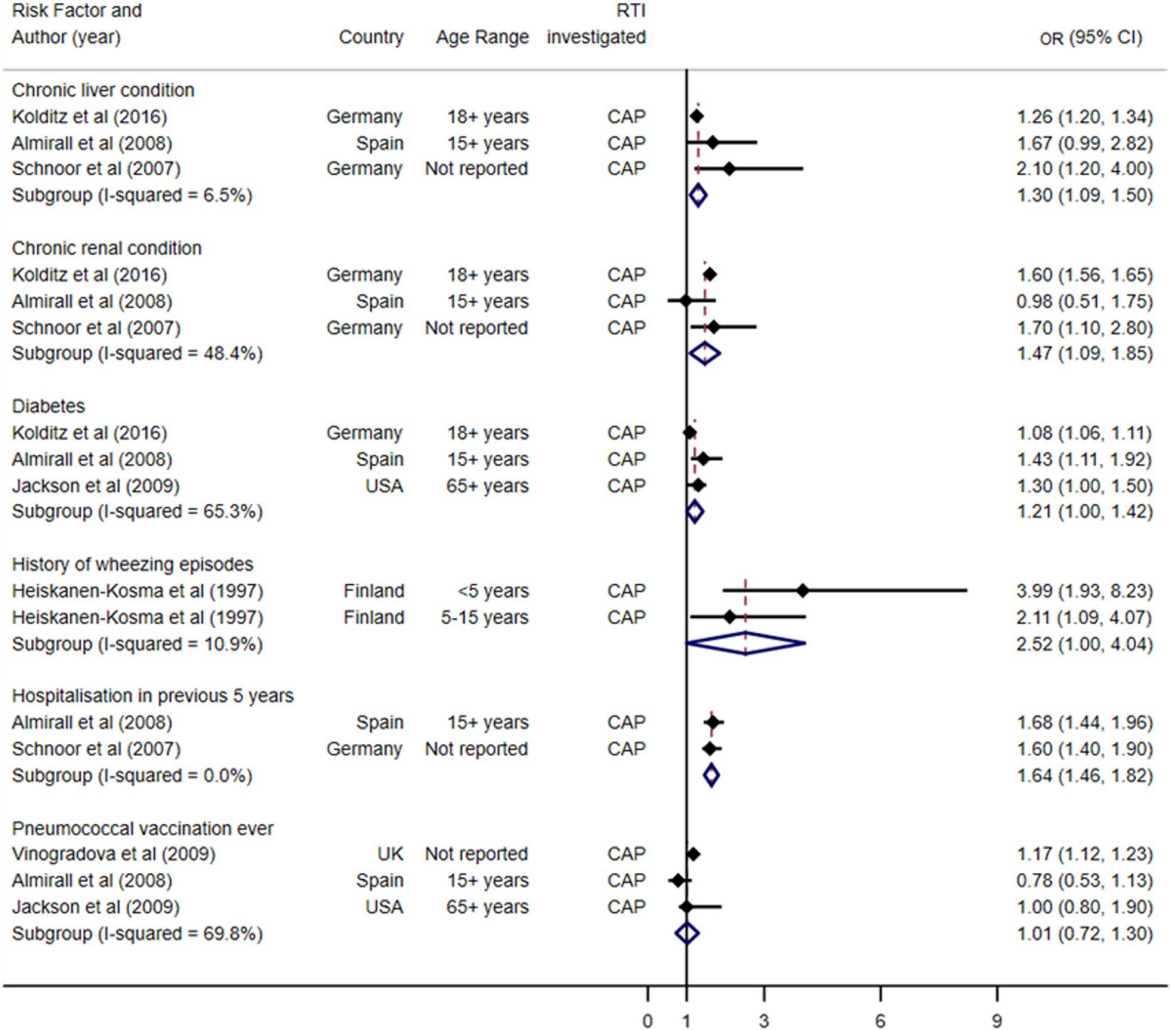
Pooled health-related risk factors for acquisition of respiratory tract infections, grouped by condition and ordered by increasing standard error

**Fig. 3:**
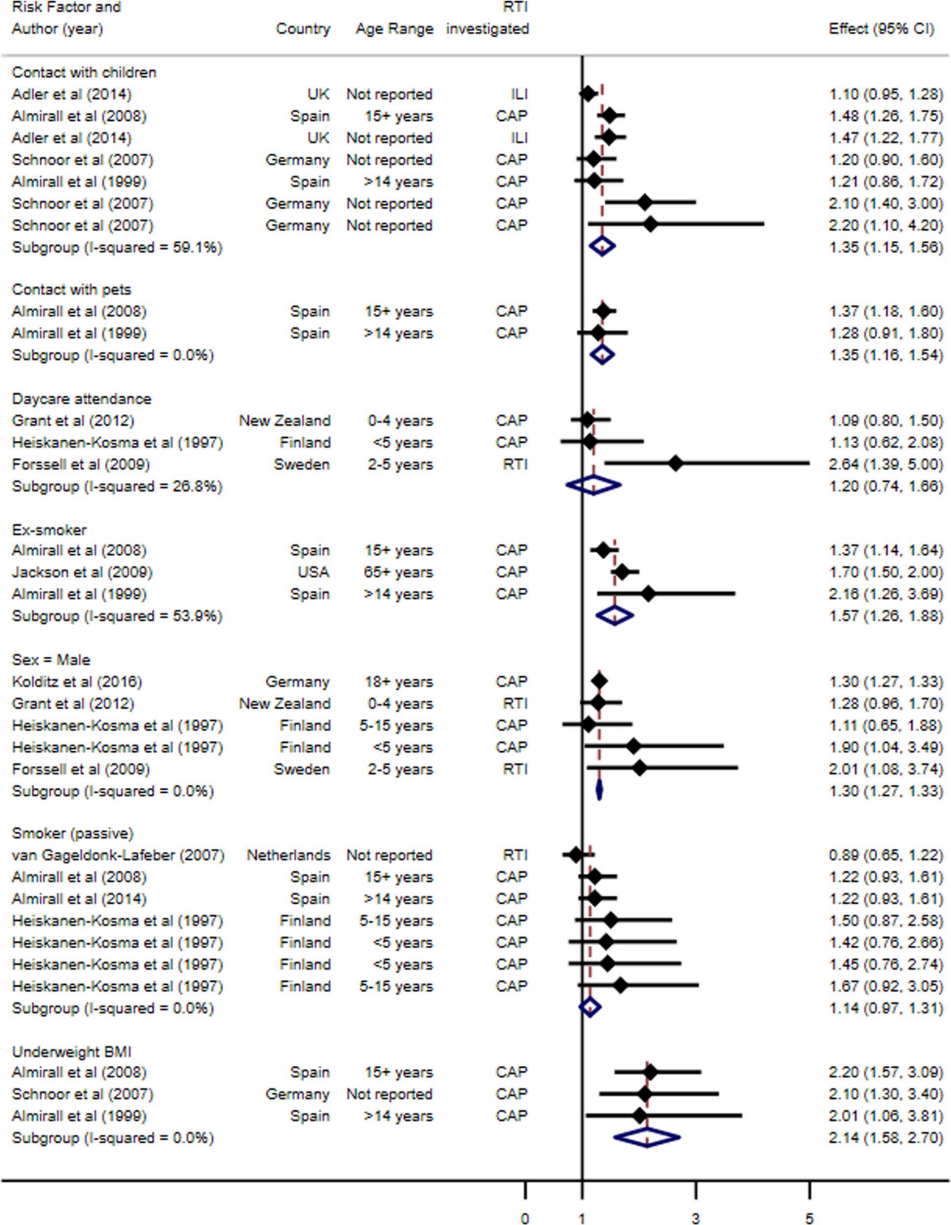
Pooled demographic, lifestyle and social risk factors for acquisition of respiratory tract infections, grouped by condition and ordered by increasing standard error

Figures 2 and 3 show the forest plot of the pooled estimates which did not have I^2^ values over the recommended 75% threshold. Figure 2 summarises the pooled health-related risk factors, and Fig. 3 summarises the pooled demographic, lifestyle and social risk factors. Studies were grouped by the risk factor investigated, most of which were exploring risk of CAP acquisition. For health-related risk factors (Fig. 2), all studies included in the meta-analysis were investigating acquisition of CAP. Chronic liver condition (pooled OR 1.30, 95% CI 1.09 to 1.50, p-value 0.343), chronic renal condition (pooled OR 1.47, 95% CI 1.09 to 1.85, p-value 0.144) and hospitalisation in previous five years (pooled OR 1.64, 95% CI 1.46 to 1.82, p-value 0.664) all suggested an increased likelihood of acquiring CAP.

For demographic, lifestyle and social risk factors (Fig. 3), the risk factor with the greatest pooled OR for RTI acquisition was an underweight BMI (pooled OR 2.14, 95% CI 1.58 to 2.70, I^2^ 0%, p-value 0.969). Male sex (pooled OR 1.30, 95% CI 1.27 to 1.33, p-value 0.661), contact with pets (pooled OR 1.35, 95% CI 1.16 to 1.54, p-value 0.720) and contact with children (pooled OR 1.35, 95% CI 1.15 to 1.56, p-value 0.053) all increased the likelihood of RTI acquisition. No associations were found for daycare attendance and passive smoking. Ex-smokers were found to have an increased risk of acquiring a RTI (pooled OR 1.57, 95% CI 1.26 to 1.88, p-value 0.114).

For the contact with children, Adler et al. (2014) is included twice. The first entry shows the OR for those who live with children; the second entry shows the reported OR for those who are in contact with children. Similarly, Schnoor et al. (2007) appears three times under contact with children. The first entry reports the ORs for those who live with only one child, the second for those who live with two children, and the third for those who live with three or more children. For both studies, these predisposing factors did not overlap and were therefore both included in our analysis under the contact with children predisposing factor.

### Publication bias

There were too few studies for each estimate measured to adequately assess publication bias.

## Discussion

### Principal findings

Our rigorously conducted systematic review meta-analysis suggests chronic liver and renal conditions, previous hospitalisation, low BMI, male sex, and contact with children and pets all increase the likelihood of people living in OECD member countries acquiring a respiratory infection.

### Strengths and limitations

To our knowledge, this is the first systematic review to identify the role of chronic liver and renal conditions, any hospitalisation in previous five years, and underweight BMI on acquisition of community-acquired respiratory tract infections, with important implications for individuals and policy makers. We are aware of four main limitations. First, some risk factors (e.g. alcohol consumption, environmental exposures, education and socioeconomic status) were measured and reported in different ways, preventing meta-analysis. It was therefore difficult to draw firm conclusions about their role and many therefore warrant further investigation. Second, most of the studies investigated community acquired pneumonia leaving an absence of evidence for less severe RTIs. It might be that our review did not capture some risk factors which may be more specific to certain RTIs, such as influenza or upper RTIs, or bacterial versus viral RTIs. Finally, we did not include studies investigating risk factors for COVID-19 because we considered it still too early in the pandemic for the evidence to have accumulated, and that this warrants a separate review. However, many of the factors we identified could be applicable to COVID-19 acquisition and should be included in future COVID-19, and other, studies.

### Results in the context of existing research

Despite the fact that respiratory infections are the single most common infections seen in the community, and the most common infection worldwide, associated with significant morbidity and unnecessary antibiotic use, there is limited evidence to date around how we might prevent them occurring in the first instance. The World Health Organisation (WHO) Global Action Plan on Antimicrobial Resistance outlines five strategic goals, one of which is to reduce the incidence of infection [41]. In order to reduce the incidence of infection it is imperative that we better understand what factors might increase our risk of acquiring common infections.

We found associations between having an underweight BMI and increased likelihood of acquiring CAP. Recent studies of BMI and risk of infection suggest both underweight and obese populations are at increased risk of acquiring infections, including respiratory and surgical site infections [42]. It might be however, that confounding is an important factor here, given that other factors including malnutrition, and co-morbidities could be influencing this association. Despite evidence of an association between underweight BMI and increased likelihood of acquiring a RTI, current National Institute for Health and Care Excellence (NICE) guidelines used by clinicians and practitioners in the management of respiratory tract infections do not highlight this [43]. Overall this review suggests that underweight BMI is associated with an increased likelihood of acquiring a RTI, however given the small number of studies which have investigated this, future research should focus on confirming this relationship in a large cohort study.

Contact with children and contact with pets were also both associated with an increased likelihood of acquiring CAP. Previous studies have shown that children are key transmitters of infection within populations [44], therefore we might expect that frequent contact with children would be a notable risk factor. Regarding contact with pets, although there is evidence this can increase the risk of transmission of infections, these are most commonly parastic and gastrointestinal infections [45]. There is currently limited evidence regarding the transmission of respiratory infections between pets and humans. One study in Austria characterised *Streptococcus pneumoiae* (the most common cause of community-acquired pneumonia) isolates from companion animals and found two sequence types known to cause invasive disease in humans also circulate in dogs [46].

We also found evidence that male children are more likely to acquire community-acquired RTIs than female children. This risk factor has also been identified in adults for COVID-19, though the reasons why males are at greater risk of respiratory infection acquisition are unclear. It is likely that sex hormones which play a role in the regulation of the immune system may be implicated in the sex differences we have observed [47].

Several previously conducted systematic reviews have investigated specific predisposing factors for acquiring RTIs in the community, including the role of Vitamin D [15, 16]. These systematic reviews explored Vitamin D supplementation on RTI incidence and Vitamin D concentrations in children with and without RTI. The findings from both studies were inconclusive, with some suggestion that Vitamin D supplementation reduced the odds of acquiring a community RTI. The high levels of heterogeneity mean these findings must be interpreted with caution. Another previous systematic review explored the effectiveness of exercise on the indicence and severity or RTIs in the community, again with inconclusive results due to small sample sizes and high levels of heterogeneity [17].

### Clinical, policy and research implications

This systematic review identifies several modifiable factors which an individual might consider in relation to their personal likelihood of acquiring RTIs, and which clinicians, policy makers and those responsible for antimicrobial stewardship might wish to consider in relation to public health messaging, either stand-alone in relation to infection acquisition, or as part of existing public health measures. Interventions known to reduce the spread of RTI, such as handwashing [48], could be targeted at ‘at risk’ groups such as ex-smokers, people with chronic liver disease, and people who work with children and animals. Researchers may wish to consider measuring the factors identified in this review in future ‘risk factor’ as well as intervention studies.

## Conclusions

We identified several risk factors associated with increased likelihood of individuals acquiring RTIs in the community, including chronic liver disease, low BMI, and contact with children and pets. Modification of risk factors and targeting prevention to vulnerable groups could reduce morbidity, mortality and antibiotic use associated with RTI, the most common type of infection worldwide.

## Supplementary Information

**Additional file 1: Table S1.** Medline and Embase search strategy (searched 20th July 2020). **Table S2.** Population, Intervention, Comparator, Outcome and Study design criteria for inclusion and exclusion. **Table S3.** Study characteristics for all included risk factor papers (n=23).

**Additional file 2: Figure S1.** Quality assessment charts for risk factor studies included in the review. **Figure S2.** Non-pooled demographic risk factor data from included studies. **Figure S3.** Non-pooled environmental risk factor data from included studies. **Figure S4.** Non-pooled lifestyle and health related risk factor data from included studies measured using non-odds ratio estimates. **Figure S5.** Non-pooled lifestyle-related risk factor data from included studies. **Figure S6.** Non-pooled social risk factor data from included studies. **Figure S7.** Non-pooled health condition-related risk factor data from included studies. **Figure S8.** Non-pooled medical history-related risk factor data from included studies.

## Data Availability

The datasets used and/or analysed during the current study are available from the corresponding author on reasonable request.

## References

[1] British Thoracic Society. The Burden of Lung Disease. London, 2006.

[2] Gulliford M, Latinovic R, Charlton J (2009). Selective decrease in consultations and antibiotic prescribing for acute respiratory tract infections in UK primary care up to 2006. J Public Health.

[3] Office for National Statistics. Sickness Absence in the Labour Market UK2012 http://webarchive.nationalarchives.gov.uk/20160105160709/http://www.ons.gov.uk/ons/dcp171776_265016.pdf.

[4] Comer M. Sickness absence in the UK labour market: 2016. In: Statistics OfN, ed., 2017.

[5] Jin X, Ren J, Li R (1990). Global burden of upper respiratory infections in 204 countries and territories, from 1990 to 2019. Lancet.

[6] Eccles R (2005). Understanding the symptoms of the common cold and influenza. Lancet Infect Dis.

[7] Public Health England (2015). English surveillance programme for antimicrobial utilisation and resistance (ESPAUR) 2010 to 2014.

[8] Lindbaek M (2006). Prescribing antibiotics to patients with acute cough and otitis media. Br J Gen Pract.

[9] Little P, Stuart B, Moore M (2013). Amoxicillin for acute lower-respiratory-tract infection in primary care when pneumonia is not suspected: a 12-country, randomised, placebo-controlled trial. Lancet Infect Dis.

[10] Llor C, Moragas A, Bayona C (2013). Efficacy of anti-inflammatory or antibiotic treatment in patients with non-complicated acute bronchitis and discoloured sputum: randomised placebo controlled trial. BMJ.

[11] World Health Organization. WHO Coronavirus Disease (COVID-19) Dashboard 2020 https://covid19.who.int/ Accessed 17 Aug 2020.

[12] Boutayeb A. The Burden of Communicable and Non-Communicable Diseases in Developing Countries. New York; 2010.

[13] Higgins JPT, Green S. Cochrane Handbook for Systematic Reviews of Interventions Version 5.1.0 [updated March 2011]. The Cochrane Collaboration, 2011. www.handbook-cochrane.org, 2011.

[14] Moher D, Liberati A, Tetzlaff J (2009). Preferred reporting items for systematic reviews and meta-analyses: the PRISMA statement. Ann Intern Med.

[15] Bergman P, Lindh AU, Bjorkhem-Bergman L (2013). Vitamin D and respiratory tract infections: a systematic review and meta-analysis of randomized controlled trials. PLoS ONE.

[16] Zisi D, Challa A, Makis A (2019). The association between vitamin D status and infectious diseases of the respiratory system in infancy and childhood. Hormones.

[17] Grande AJ, Eogh J, Silva V (2020). Exercise versus no exercise for the occurrence, severity, and duration of acute respiratory infections. Cochrane Database Syst Rev.

[18] Kolditz M, Tesch F, Mocke L (2016). Burden and risk factors of ambulatory or hospitalized CAP: a population based cohort study. Respir Med.

[19] Harpsoe MC, Nielsen NM, Friis-Moller N (2016). Body mass index and risk of infections among women in the Danish National Birth Cohort. Am J Epidemiol.

[20] Beamer PI, Lothrop N, Lu Z (2016). Spatial clusters of child lower respiratory illnesses associated with community-level risk factors. Pediatr Pulmonol.

[21] Adler AJ, Eames KTD, Funk S (2014). Incidence and risk factors for influenza-like-illness in the UK: online surveillance using Flusurvey. BMC Infect Dis.

[22] Blumentals WA, Nevitt A, Peng MM (2012). Body mass index and the incidence of influenza-associated pneumonia in a UK primary care cohort. Influenza Other Respir Viruses.

[23] Gessner BD, Chimonas MA, Grady SC (2010). It takes a village: community education predicts paediatric lower-respiratory infection risk better than maternal education. J Epidemiol Commun Health.

[24] Brownstein JS, Mandl KD (2008). Pediatric population size is associated with geographic patterns of acute respiratory infections among adults. Ann Emerg Med.

[25] Neuman MI, Willett WC, Curham GC (2007). Vitamin and micronutrient intake and the risk of community-acquired pneumonia in US women. Am J Med.

[26] Schnoor M, Klante T, Beckmann M (2007). Risk factors for community-acquired pneumonia in German adults: the impact of children in the household. Epidemiol Infect.

[27] de Roux A, Cavalcanti M, Marcos MA (2006). Impact of alcohol abuse in the etiology and severity of community-acquired pneumonia. Chest.

[28] Iversen L, Hannaford PC, Price DB (2005). Is Living in a Rural Area Good for Your Respiratory Health?. Chest.

[29] Forssell G, Hakansson A, Mansson NO (2009). Risk factors for respiratory tract infections in children aged 2–5 years. Scand J Prim Health Care.

[30] Huijskens EG, Smit LA, Rossen JW (2016). Evaluation of patients with community-acquired pneumonia caused by zoonotic pathogens in an area with a high density of animal farms. Zoonoses Public Health.

[31] Almirall J, Serra-Prat M, Bolibar I (2014). Passive smoking at home is a risk factor for community-acquired pneumonia in older adults: a population-based case-control study. BMJ Open.

[32] Dublin S, Jackson ML, Nelson JC (2009). Statin use and risk of community acquired pneumonia in older people: population based case-control study. BMJ.

[33] Grant CC, Emery D, Milne T (2012). Risk factors for community-acquired pneumonia in pre-school-aged children. J Paediatr Child Health.

[34] Vinogradova Y, Hippisley-Cox J, Coupland C (2009). Identification of new risk factors for pneumonia: population-based case–control study. Br J Gen Pract.

[35] Jackson ML, Nelson JC, Jackson LA (2009). Risk factors for community-acquired pneumonia in immunocompetent seniors. J Am Geriatr Soc.

[36] Almirall J, Bolibar I, Serra-Prat M (2008). New evidence of risk factors for community-acquired pneumonia: a population-based study. Eur Respir J.

[37] Almirall J, Bolibar I, Balanzo X (1999). Risk factors for community-acquired pneumonia in adults: a population-based case-control study. Eur Respir J.

[38] Heiskanen-Kosma T, Korppi M, Jokinen C (1997). Risk factos for community-acquired pneumonia in children: a population-based case–control study. Scand J Infect Dis.

[39] van Gageldonk-Lafeber AB, van der Sande MAB, Heijnen LA (2007). Risk factors for acute respiratory tract infections in general practitioner patients in The Netherlands: a case–control study. BMC Infect Dis.

[40] Maccioni L, Weber S, Elgizouli M (2018). Obesity and risk of respiratory tract infections: results of an infection-diary based cohort study. BMC Public Health.

[41] Organisation WH. Global Action Plan on Antimicrobial Resistance, 2015.

[42] Dobner J, Kaser S (2018). Body mass index and the risk of infection - from underweight to obesity. Clin Microbiol Infect.

[43] National Institute for Health and Care Excellence (NICE). Respiratory tract infections (self-limiting): prescribing antibiotics. 2008; https://www.nice.org.uk/guidance/cg69/resources/respiratory-tract-infections-selflimiting-prescribing-antibiotics-pdf-975576354757 accessed 3 Sept 2020. 31815394

[44] Bryce A, Costelloe C, Hawcroft C (2016). Faecal carriage of antibiotic resistant *Escherichia coli* in asymptomatic children and associations with primary care antibiotic prescribing: a systematic review and meta-analysis. BMC Infect Dis.

[45] O'Neil J (2018). Zoonotic infections from common household pets. J Nurse Pract.

[46] Ginders M, Leschnik M, Kunzel F (2017). Characterization of *Streptococcus pneumoniae* isolates from Austrian companion animals and horses. Acta Vet Scand.

[47] Kadel S, Kovats S (2018). Sex hormones regulate innate immune cells and promote sex differences in respiratory virus infection. Front.

[48] Little P, Stuart B, Hobbs FDR (2015). An internet-delivered handwashing intervention to modify influenza-like illness and respiratory infection transmission (PRIMIT): a primary care randomised trial. Lancet.

