# Supplementary material for: Predisposing factors to acquisition of acute respiratory tract infections in the community: a systematic review and meta-analysis
Source: BMC Infect Dis. 2021 Dec 14;21:1254. doi: 10.1186/s12879-021-06954-3 (PMC8670045; doi:10.1186/s12879-021-06954-3)
Supplement: Supplementary file 1 — Additional file 1: Table S1. Medline and Embase search strategy (searched 20th July 2020). Table S2. Population, Intervention, Comparator, Outcome and Study design criteria for inclusion and exclusion. Table S3. Study characteristics for all included risk factor papers (n=23). [file 12879_2021_6954_MOESM1_ESM.docx]

# Supplementary Tables

Table 1. Medline and Embase search strategy (searched 20^th^ July 2020)

| 1. Exp Respiratory Tract Infections/ | 29. High temperature.mp |
| --- | --- |
| 1. Respiratory tract infection$.mp | 30. Acutely ill.mp |
| 1. Respiratory infection$.mp | 31. Acute illness$.mp |
| 1. RTI$.tw | 32. Tonsillopharyngitis.tw |
| 1. URTI$.tw | 33. Self-limiting.tw |
| 1. LRTI$.tw | 34. Self-limiting.mp |
| 1. Chest infection$.mp | 35. Transmission.tw |
| 1. Bronchitis.tw | 36. Exp Communicable Diseases/ |
| 1. Bronchiolitis.tw | 37. 1 or 2 or 3 or 4 or 5 or 6 or 7 or 8 or 9 or 10 or 11 or 12 or 13 or 14 or 15 or 16 or 17 or 18 or 19 or 20 or 21 or 22 or 23 or 24 or 25 or 26 or 27 or 28 or 29 or 30 or 31 or 32 or 33 or 34 or 35 or 36 |
| 1. Pneumonia.tw | 38. Exp Community-Acquired Infections/ |
| 1. Croup.tw | 39. Community-acquired.mp |
| 1. Sore throat.mp | 40. Community.tw |
| 1. Tonsilitis.tw | 41. Family practice.mp |
| 1. Pharyngitis.tw | 42. Exp Primary Health Care/ |
| 1. Nasopharyngitis.tw | 43. Primary care.mp |
| 1. Laryngitis.tw | 44. Ambulatory.mp |
| 1. Exp Cough/ | 45. 38 or 39 or 40 or 41 or 42 or 43 or 44 |
| 1. Exp Respiration Disorders/ | 46. Exp Risk Factors/ |
| 1. Tracheitis.tw | 47. Risk.mp |
| 1. Sinusitis.tw | 48. Predispose.tw |
| 1. Rhino-sinusitis.tw | 49. Identif$.tw |
| 1. Cough.tw | 50. Symptom$.tw |
| 1. Common cold.mp | 51. Sign$.tw |
| 1. Exp Influenza.tw | 52. Predict$.mp |
| 1. Flu.tw | 53. Associat$.mo |
| 1. Exp Fever/ | 54. 46 or 47 or 48 or 49 or 50 or 51 or 52 or 53 |
| 1. Fever.tw | 55. 37 and 45 and 54 |
| 1. Febrile.tw |  |

Table 2. Population, Intervention, Comparator, Outcome and Study design criteria for inclusion and exclusion

| **PICOS** | **Inclusion criteria** | **Exclusion criteria** |
| --- | --- | --- |
| **Population** | Humans only, no age restrictions  Patients visiting primary care centre with symptoms of an acute RTI:   - Upper (including common cold, tonsillitis, sinusitis, laryngitis) - Lower (including pneumonia, bronchitis, bronchiolitis) - Influenza   Patients recruited from the community, e.g. household recruitment or community groups  Patients recruited on admission to hospital with a community-acquired RTI (e.g. community-acquired pneumonia)  Studies must be conducted in OECD member countries  Patients should be largely free from any co-morbidities which may increase their likelihood of acquiring an RTI (e.g. chronic obstructive pulmonary disease, cystic fibrosis, HIV). | Not conducted in an OECD member country  Study participants have chronic or long-term RTIs (including COPD)  Patients have hospital-acquired RTIs (including ventilator-associated pneumonia)  Study participants all have significant co-morbidities which may increase their likelihood of acquiring an RTI  Study participants are all smokers  Study participants are premature infants in neonatal unit  Study participants all have COVID-19 |
| **Intervention** | *Not applicable* | *Not applicable* |
| **Comparator** | *Not applicable* | *Not applicable* |
| **Outcomes** | Studies reporting or investigating quantitative data related to:   - Risk factors associated with the acquisition or transmission of an RTI in the community - Studies quantifying change (increase/decrease) and associated risk factors for the change in acquisition and/or transmission rates of RTIs in humans | Studies do not measure or quantify at least one of the following:   - Risk factors associated with acquisition or transmission of an RTI in the community - Change (increase/decrease) and associated risk factors for the change in acquisition and/or transmission rates of RTIs in humans |
| **Study design** | Observational studies   - Cohort - Case-control - Cross-sectional - Longitudinal   Experimental studies/trials  Epidemiological studies | Qualitative studies without any quantitative data  Systematic reviews  Studies which have been included in a previous systematic review conducted in the last 10 years investigating one specific predisposing factor to RTI acquisition, e.g. smoking.  Economic studies  Critical reviews/expert opinions without any primary data |

Table 3. Study characteristics for all included risk factor papers (n=23)

| **Ref. No.** | **Author** | **Year** | **Country** | **Study design** | **Recruitment** | **No. of study participants** | **Participant age range** | **RTI investigated** | **Risk factors investigated** | **Risk factor unit of measurement** | **Crude risk factor data available** |
| --- | --- | --- | --- | --- | --- | --- | --- | --- | --- | --- | --- |
| 39 | Maccioni et al | 2018 | Germany | Cross-sectional | Community | 1455 | 18 to 70 years | Any RTI | obese BMI | Odds ratio | Yes |
| 17 | Kolditz et al | 2016 | Germany | Observational | Emergency department | 1,837,080 | ≥18 years | CAP | male sex,  cancer,  chronic GI/liver disease,  chronic renal disease,  chronic lung disease,  diabetes mellitus | Odds ratio | Yes |
| 18 | Harpsoe et al | 2016 | Denmark | Observational | Primary care | 75,001 | Not reported (mean age 30.7 years) | Any RTI | underweight BMI,  overweight BMI,  obese BMI | Hazard ratio | Yes |
| 19 | Beamer et al | 2016 | USA | Observational | Primary care | 1246 | ≤3 years | Lower RTI | lower socioeconomic status,  poorer housing characteristics,  increased air pollution | Odds ratio | Yes |
| 29 | Huijskens et al | 2016 | Netherlands | Case-control | Emergency department | 1504 | 20 to 94 years | CAP | one or more farms within 1km of home,  presence of swine within 1km of home,  presence of poultry within 1km of home,  presence of cattle within 1km of home,  presence of goats within 1km of home,  presence of sheep within 1km of home | Odds ratio | Yes – but no comparable data for meta-analysis |
| 30 | Almirall et al | 2014 | Spain | Case-control | Primary care | 1003 | >14 years | CAP | Passive tobacco consumption at home (all ages),  Passive tobacco consumption at home (<65 years);  Passive tobacco consumption at home (≥65 years) | Odds ratio | Yes |
| 20 | Adler et al | 2014 | UK | Observational | Community | 5943 | Not reported, all age groups included | ILI | Female sex;  Unvaccinated;  Age 18-24;  Age 25-34;  Age 35-44;  Age 45-64;  Age 65+;  Contact with children;  Live with children;  Smoker;  Take public transport;  Underlying health condition;  Employment status | Odds ratio | Yes |
| 21 | Blumentals et al | 2012 | UK | Observational | Primary care | 1,074,315 | ≥18 years | CAP | Underweight BMI;  Overweight BMI;  Obsess BMI;  Type II diabetes;  Hypertension;  Statin use;  Antibiotic use;  Current or ex-smoker;  Influenza vaccination | Incidence rate ratio | Yes |
| 32 | Grant et al | 2012 | New Zealand | Case-control | Emergency department | 1012 | 0 to 4 years | CAP | Male sex;  Age in years;  Birthweight <2.5kg;  Maori ethnicity;  Pacific ethnicity;  Not breastfed;  0 to 1 months exclusive breastfeeding;  2 months exclusive breastfeeding;  Not fully immunised;  Less than 30 min outside in past 4 weeks;  Attends daycare;  Previous RTIs;  Asthma in previous year;  Maternal cough most days;  Maternal history of pneumonia;  Siblings hospitalised for pneumonia | Odds ratio | Yes |
| 22 | Gessner et al | 2010 | USA | Observational | Primary care | 17,913 | <2 years | Lower RTIs | 1 to 2 years postsecondary maternal education;  High school graduate;  Some high school;  Proportion of adults living in child’s community with <7 years formal education (10-20%);  Proportion of adults living in child’s community with <7 years formal education (>20%); | Odds ratio | Yes |
| 31 | Dublin et al | 2009 | USA | Case-control | Primary care | 3360 | 65 to 94 years | CAP | Current statin use | Odds ratio | Yes |
| 28 | Forssell et al | 2009 | Sweden | Cohort | Community | 190 | 2 to 5 years | Any RTI | Age, male sex, low birthweight, breastfeeding, atopy, siblings, smoking, daycare attendance | Odds ratio | Yes |
| 34 | Jackson et al | 2009 | USA | Case-control | Outpatient/Inpatient | 3519 | >65 years | CAP | Lung disease;  Previous pneumonia;  Former smoker;  Current smoker;  Heart disease;  Diabetes;  Dementia;  History of stroke;  Blindness or vision impairment;  Lowest quintile of sex-specific weight;  Second quintile of sex-specific weight;  Fourth quintile of sex-specific weight;  Highest quintile of sex-specific weight;  Current alcoholism;  History of Parkinson’s disease;  Pneumococcal vaccine received ever;  Influenza vaccine received ever | Odds ratio | Yes |
| 33 | Vinogradova et al | 2009 | UK | Case-control | Primary care | 88,571 | Not reported, all age groups included | CAP | Any cancer;  Dementia;  Influenza vaccination in previous 12 months;  Parkinson’s disease;  Stroke or TIA;  Smoker;  Pneumococcal vaccination in previous 60 months;  Townsend score;  Rheumatoid arthritis;  Multiple sclerosis;  Osteoporosis;  osteosrthritis | Odds ratio | Yes |
| 35 | Almirall et al | 2008 | Spain | Case-control | Primary care | 2662 | >14 years | CAP | Cancer;  Chronic liver disease;  Chronic renal failure;  Dementia;  Diabetes mellitus;  Hospital admission in previous 5 years;  Influenza vaccine in previous year;  Parkinson’s disease;  Stroke or TIA;  Obese BMI;  Overweight BMI;  Underweight BMI;  Smoker;  Contact with any pets;  Usual contract with children;  Chronic bronchitis;  Pneumococcal vaccine ever;  Previous RTIs;  Work-related exposures;  Temperature changes at work;  >10 persons at home;  Middle educational level;  High educational level;  Single,widowed or diverced;  Heart failure;  Heart valve disease;  Coronary artery disease;  Asthma;  Nonactive pulmonary tuberculosis;  Epilepsy;  Phychiatric disorders;  Gastro-oesophageal reflux;  HIV;  Visit to dentist in previous month;  Previous antibiotic use;  H. influenzae type B vaccine ever | Odds ratio | Yes |
| 23 | Brownstein et al | 2008 | USA | Observational | Outpatient/inpatient | 157,542 | ≥18 years | Any RTI | Effect of % increase in paediatric population on rate of adult acute RTIs (per 1000 population) | Risk ratio | Yes, but no comparable data for meta-analysis |
| 24 | Neuman et al | 2007 | USA | Observational | Primary care | 83,165 | 27 to 44 years | CAP | Multivitamin intake | Risk ratio | Yes |
| 25 | Schnoor et al | 2007 | Germany | Observational | Primary care | 1137 | Not reported, all age groups included | CAP | Chronic liver disease;  Chronic renal disease;  Chronic pulmonary disease;  Chronic heart disease;  Hospitalisation in previous 5 years;  Obese BMI;  Overweight BMI;  Underweight BMI;  Ever smoked;  Contact with children in household;  Previous RTIs;  History of CAP;  Education | Odds ratio | Yes |
| 38 | van Gageldonk-Lafeber | 2007 | Netherlands | Case-control | Primary care | 986 | All ages | Acute RTI | Exposure to respiratory complaints within/outside household; number of children in household; children in primary/secondary school; dampness or mould at home; mechanical ventilation system; smoking; passive smoking | Odds ratio | Yes |
| 26 | de Roux et al | 2006 | Spain | Observational | Outpatient/inpatient | 1347 | Not included, mean age 68 years | CAP | Alcoholism | Odds ratio | Yes |
| 27 | Iversen et al | 2005 | UK | Observational | Primary care | 170 | ≥16 years | CAP | Rural/urban | Odds ratio | Yes |
| 36 | Almirall et al | 1999 | Spain | Case-control | Primary care | 680 | >14 years | CAP | Obese BMI;  Overweight BMI;  Underweight BMI;  Ex-smoker;  Contact with pets;  Contact with children;  Alcohol intake | Odds ratio | Yes |
| 37 | Heiskanen-Kosma et al | 1997 | Finland | Case-control | Primary care | 451 | ≤15 years | CAP | Recurrent RTIs;  History of wheezing episodes | Odds ratio | Yes |

Where RTI = respiratory tract infection; ILI = influenza-like-illness; CAP = community-acquired pneumonia
