# Supplementary material for: Predisposing factors to acquisition of acute respiratory tract infections in the community: a systematic review and meta-analysis
Source: BMC Infect Dis. 2021 Dec 14;21:1254. doi: 10.1186/s12879-021-06954-3 (PMC8670045; doi:10.1186/s12879-021-06954-3)
Supplement: Supplementary file 2 — Additional file 2: Figure S1. Quality assessment charts for risk factor studies included in the review. Figure S2. Non-pooled demographic risk factor data from included studies. Figure S3. Non-pooled environmental risk factor data from included studies. Figure S4. Non-pooled lifestyle and health related risk factor data from included studies measured using non-odds ratio estimates. Figure S5. Non-pooled lifestyle-related risk factor data from included studies. Figure S6. Non-pooled social risk factor data from included studies. Figure S7. Non-pooled health condition-related risk factor data from included studies. Figure S8. Non-pooled medical history-related risk factor data from included studies. [file 12879_2021_6954_MOESM2_ESM.docx]

# Supplementary Figures

Figure 1. Quality assessment charts for risk factor studies included in the review


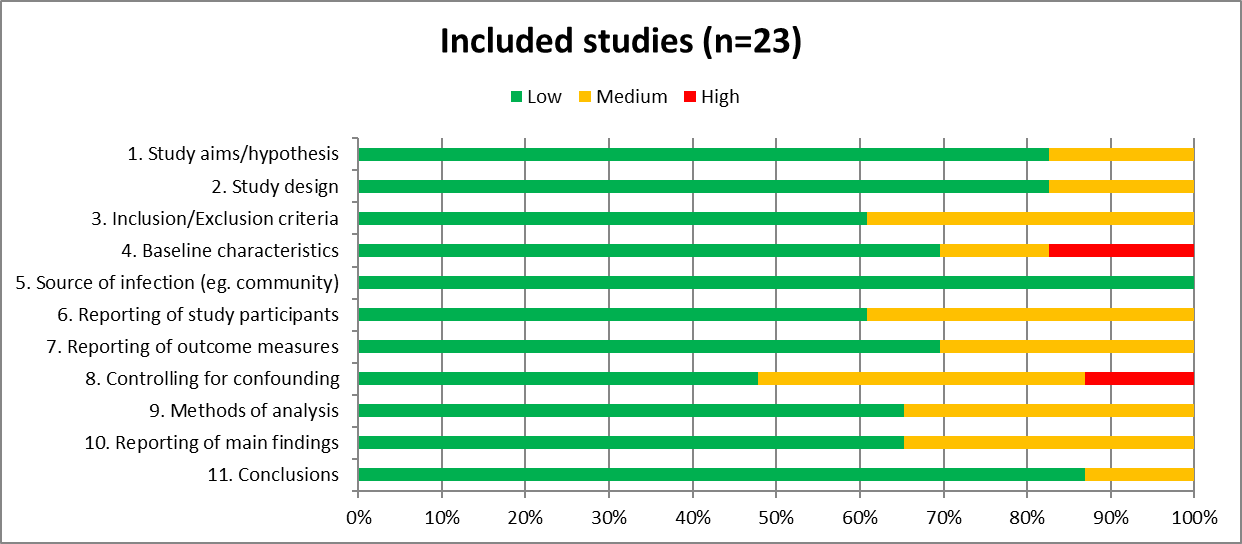


Figure 2. Non-pooled demographic risk factor data from included studies


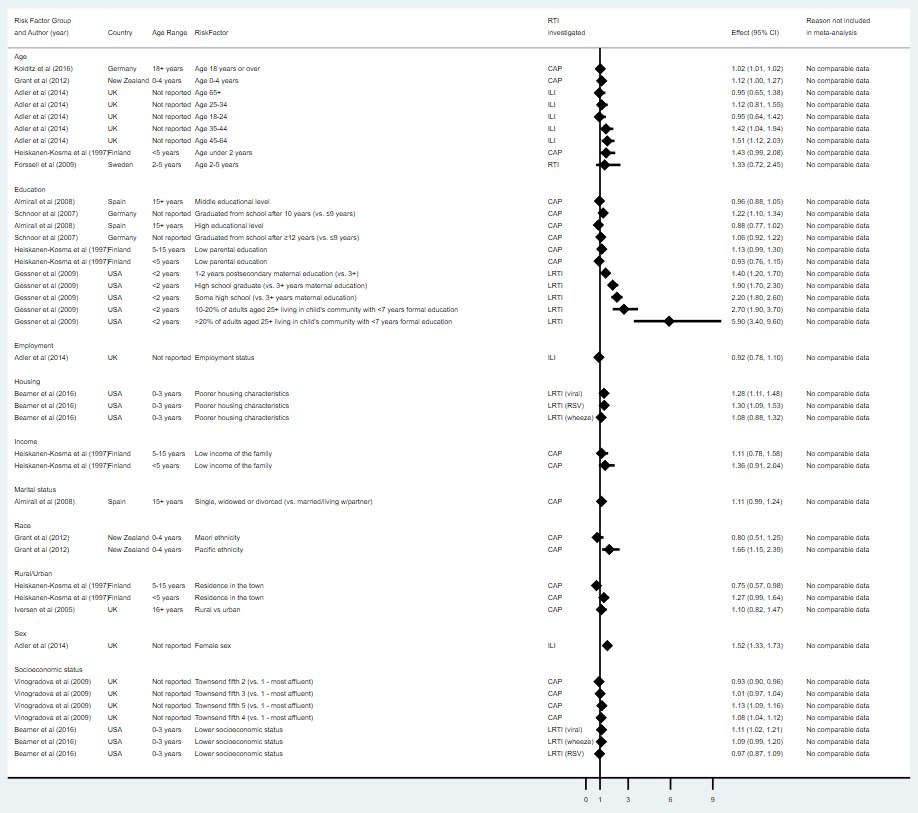


Where OR = odds ratio; 95% CI = 95% confidence interval; CAP = community-acquired pneumonia; ILI = influenza-like-illness; LRTI = lower respiratory tract infection; RSV = respiratory syncytial virus

Figure 3. Non-pooled environmental risk factor data from included studies

*
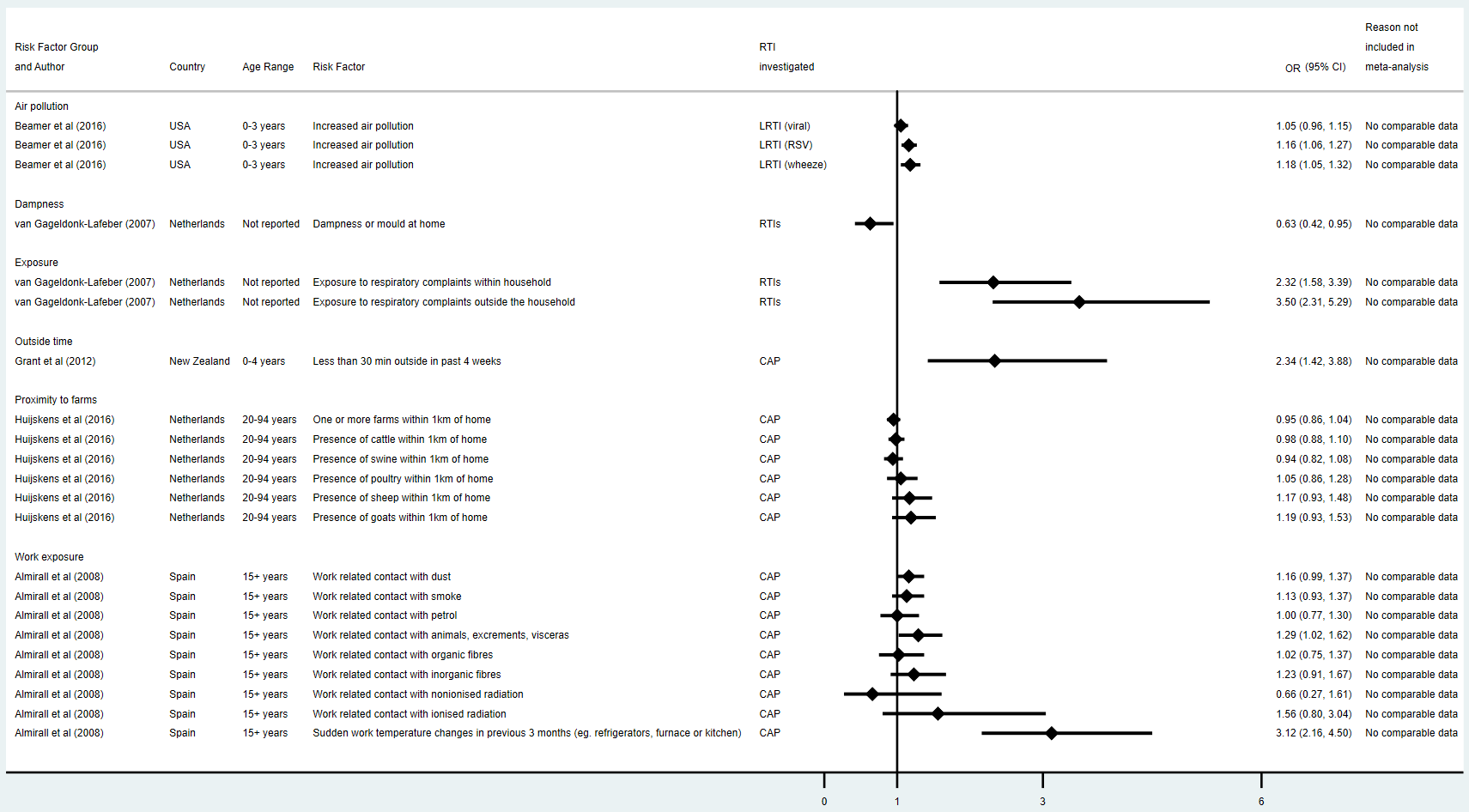
*

Where OR = odds ratio; 95% CI = 95% confidence interval; CAP = community-acquired pneumonia; LRTI = lower respiratory tract infection; RSV = respiratory syncytial virus; RTI = respiratory tract infection

Figure 4*.* Non-pooled lifestyle and health related risk factor data from included studies measured using non-odds ratio estimates


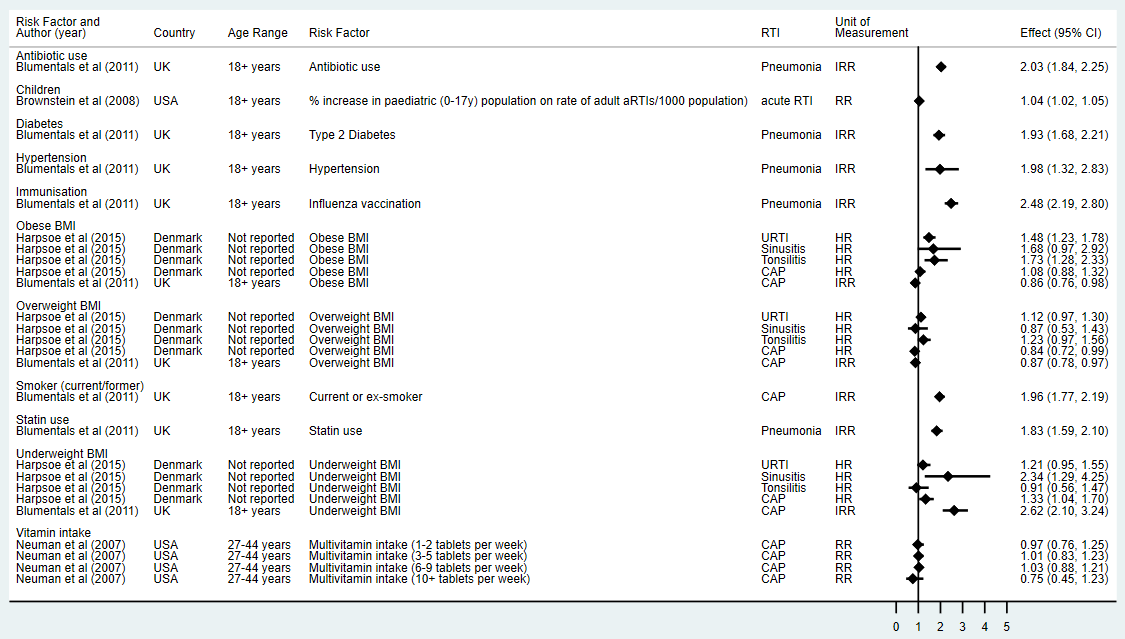


Where BMI = Body Mass Index; URTI = upper respiratory tract infection; CAP = community-acquired pneumonia; ; IRR = incidence rate ratio; HR = hazard ratio; RR = risk ratio; ES = effect size; 95% CI = 95% confidence interval

Figure 5. Non-pooled lifestyle-related risk factor data from included studies

*
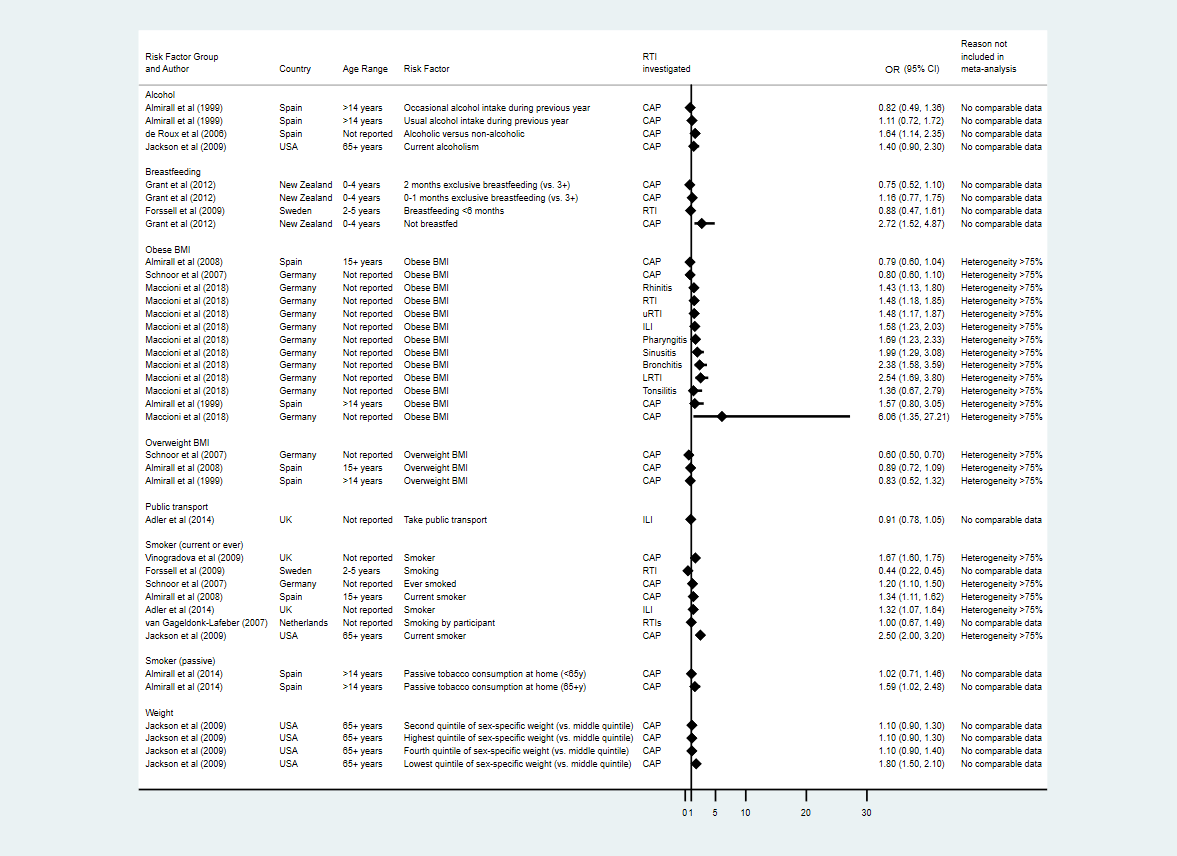
*

Where OR = odds ratio; 95% CI = 95% confidence intervals; CAP = community-acquired pneumonia; RTI = respiratory tract infection; ILI = influenza-like-illness

Figure 6. Non-pooled social risk factor data from included studies

*
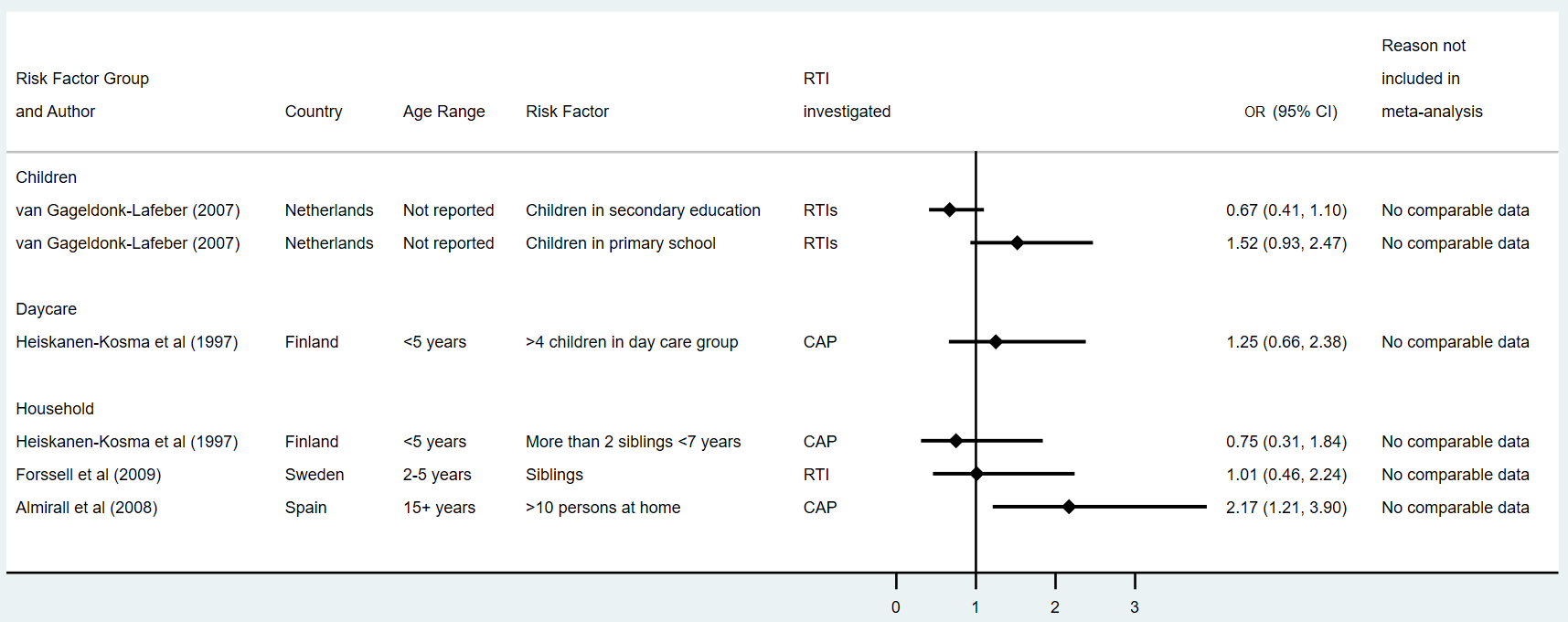
*

Where OR = odds ratio; 95% CI = 95% confidence intervals; RTI = respiratory tract infection; CAP = community-acquired pneumonia

Figure 7. Non-pooled health condition-related risk factor data from included studies

*
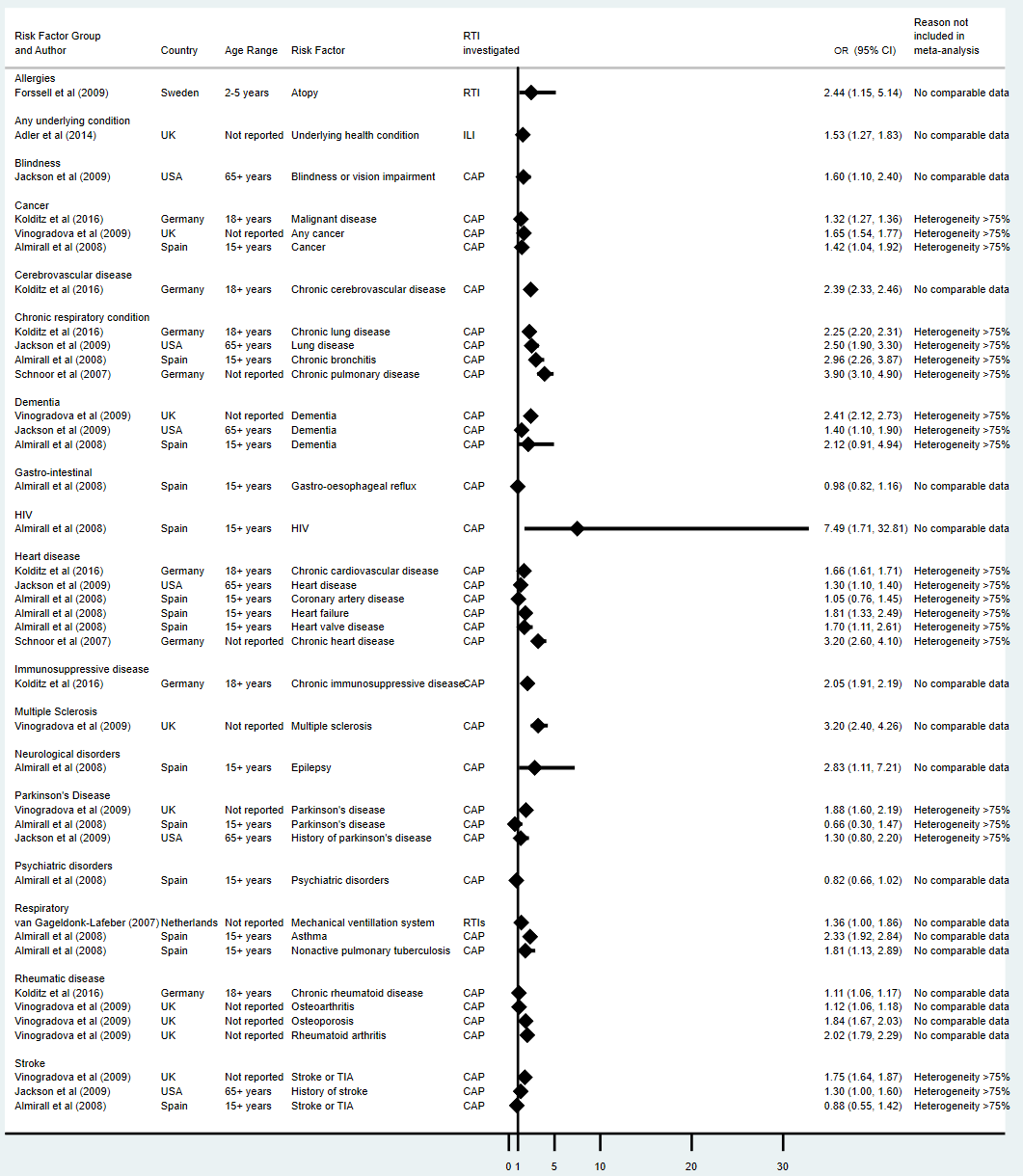
*

Where OR = odds ratio; 95% CI = 95% confidence intervals; RTI = respiratory tract infection; ILI = influenza-like illness; CAP = community-acquired pneumonia

Figure 8. Non-pooled medical history-related risk factor data from included studies


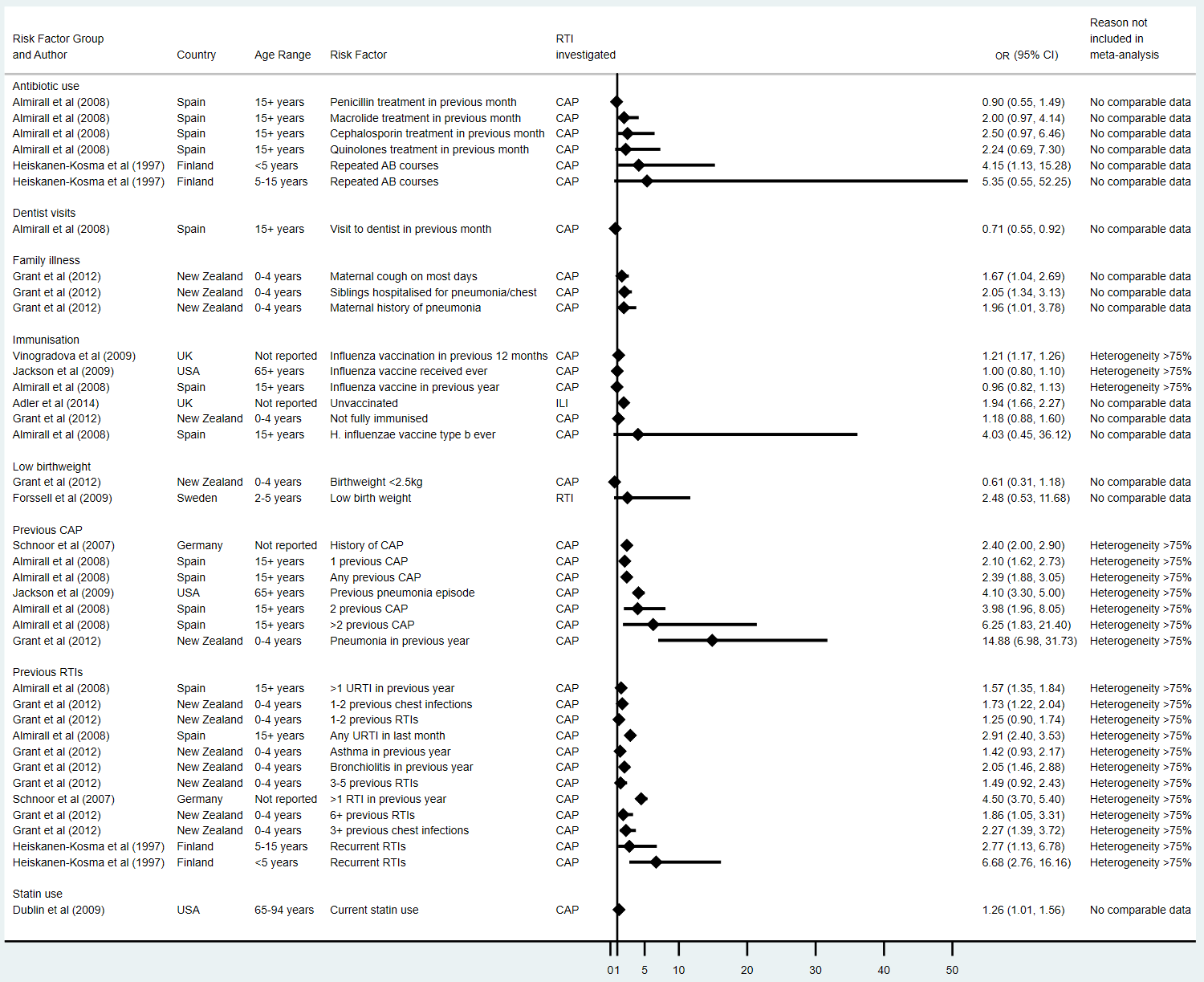


Where OR = odds ratio; 95% CI = 95% confidence intervals; CAP = community-acquired pneumonia; ILI = influenza-like illness; RTI = respiratory tract infection.
